# Rethinking the use of germline *CHEK2* mutation as a marker for PARP inhibitor sensitivity

**DOI:** 10.1093/jncics/pkae045

**Published:** 2024-07-01

**Authors:** Thomas J Hayman

**Affiliations:** Department of Therapeutic Radiology, Yale University School of Medicine, New Haven, CT, USA

Approximately 5% of patients diagnosed with breast cancer will have pathogenic or likely pathogenic germline variants in *BRCA1* or *BRCA2*. *BRCA1* and *BRCA2* are tumor suppressors that have critical roles in the regulation of homologous recombination (HR), a major error-free pathway for DNA double-strand break (DSB) repair (1-3). Defects in HR lead to genomic instability and predisposition to cancer development (4). In the context of cancer therapy, the concept of synthetic lethality has emerged because of the finding that tumors deficient in *BRCA1* or *BRCA2* exhibit an HR-deficient state (HRD) (4-6). Specifically, synthetic lethality between genes is generally defined as a phenomenon where simultaneous inactivation of 2 genes results in cell death, but individual inactivation has little effect. In this context, using poly(ADP-ribose) polymerase (PARP) inhibitors (PARPi) to generate DNA DSBs that require HR for repair can selectively target BRCA-deficient tumor cells and spare normal tissue (6). Thus, there has been significant interest in using PARPi to treat *BRCA*-mutant breast cancer in the clinic (7,8). As such, multiple trials have been conducted in patients with *BRCA*-mutated breast cancer. In the OlympiAD trial, the use of Olaparib in patients with germline BRCA-mutated HER2-negative metastatic breast cancer resulted in improved progression-free survival (PFS) (9). In the adjuvant setting, the OlympiA trial demonstrated improvements in overall survival (OS) after curative intent therapy in patients with germline pathogenic variants (gPVs) in BRCA1/2 in high-risk, early breast cancer (10,11).

Given the successes seen using PARPi in patients with *BRCA*-mutated breast cancers, there has been substantial interest in expanding the use of PARPi to the treatment of breast cancers that exhibit HRD, a phenotype referred to as “BRCAness” (4). It has been increasingly recognized that a significant proportion of sporadic and hereditary malignancies, including breast cancers, have defects in HR (4,12-14). Preclinical evidence has demonstrated roles for multiple DNA repair genes in regulating HR with defects in these proteins leading to PARPi sensitivity (4,15). These genes include *ATM* (ataxia-telangiectasia mutated), *ATR* (ataxia telangiectasia and Rad3-related), *CHEK1*, *CHEK2*, and *Rad51*, among others. Thus, multiple clinical trials have been conducted aimed at examining the role of PARPi in patients with mutations in HR-related genes. The Phase II Translational Breast Cancer Research Consortium (TBCRC) 048 enrolled 54 patients with metastatic breast cancer and mutations in HR-related genes with the primary endpoint of defining objective response rate (ORR) to PARPi (laparib) treatment (16). In total, 87% of enrolled patients had mutations in *PALB2*, somatic *BRCA1/2*, *ATM*, or *CHEK2*. Confirmed responses were seen only with germline *PALB2* mutations (ORR, 82%) or somatic *BRCA1/2* mutations (ORR, 50%). No responses were observed with *ATM* or *CHEK2* mutations. Similar results were seen in the Phase II tumor agnostic study showing no responses with olaparib treatment in patients with likely pathogenic germline or somatic *CHEK2* mutations (17). As such, these results call into question the relevance of specific mutations, such as *CHEK2* mutations, in regulating HR and thus PARPi responses.

Because of these confounding clinical results, Hinić and colleagues performed a series of bioinformatics-based experiments in patients with biallelic gPVs in *CHEK2*, aimed at understanding the impact of the gPVs on genomic markers of HR (18). They analyzed the genomes of 16 cancers from 9 individuals homozygous for the most common loss-of-function (LoF) gPV in *CHEK2* (c.1100del) (19). This gPV is a low to moderate penetrance breast cancer risk gene whose truncating mutation increases the breast cancer risk by approximately 2-fold in woman and 10-fold in men (19). They analyzed markers of genomic instability, including large-scale transitions (LST), telomeric allelic imbalances (tAI), tumor mutation burden (TMB), and mutational signatures, where the presence of LSTs and tAIs are known markers of genomic instability associated with HRD (20).

In these analyses, the authors found that none of the analyzed *CHEK2* mutated tumors had LST ≥15, which is considered the cutoff value for HRD (20). In contrast, BRCA1/2-deficient ovarian cancers presented with a median number of LSTs of 23 and elevated tAIs (median of 16 compared to median of 7 in CHEK2 mutated tumors). These results were confirmed through analyses of TCGA cohorts of patients with heterozygous gPVs in *CHEK2* or *BRCA1/2*. Similar findings were seen upon analyses of TMB where BRCA mutant tumors showed significantly higher TMB than tumors with gPVs in *CHEK2*. They then show that the minority of tumors (∼13%) with homozygous gPVs in *CHEK2* had the SBS3 HRD-related mutational signature, in contrast to 86% of the BRCA-deficient group. Intriguingly, they also demonstrate that the spectrum of mutational signatures of *CHEK2*-deficient tumors resembles that of sporadic tumors more so than what is observed in BRCA-deficient cancers.

This study presents genomic evidence from clinical specimens that homozygous LoF gPVs in *CHEK2* do not contribute to HRD, in contrast to BRCA-deficient tumors that show evidence of HRD in their analyses. The findings of this study provide a rationale for the clinical trial data demonstrating a lack of efficacy of PARPi in patients with *CHEK2* mutations. Although some preclinical data have suggested that RNA interference (RNAi) based knockdown of CHK2 protein expression leads to PARPi sensitivity (15), more recent data using CRISPR (Clustered Regularly Interspaced Short Palindromic Repeats), a technique known to have fewer off-target effects than RNAi, demonstrates that CRISPR-based knockout of *CHEK2* confers resistance to PARPi through increased expression of BRCA2 (21). Furthermore, in support of the authors’ findings, Boonen et al. showed no effects of *CHEK2* knockout on HR efficiency using the DR-GFP (direct repeatgreen fluorescent protein) assay (a commonly used preclinical assay to assess HR activity) (22). The authors’ approach coupled with negative clinical trials using PARPi in *CHEK2*-mutated cancers highlights the importance of identifying functional HRD before the use of a specific gPV as a biomarker for PARPi use.

As the authors note, there are a few limitations of the current study. Their approach uses shallow whole-genome sequencing and thus is unable to detect smaller structural variants. The analyses were also limited in terms of non-breast tumors (with only 4 non-breast origins explored) as well as in the number of tumors analyzed overall. Nonetheless, these data provide a rationale for the lack of efficacy of PARPi in *CHEK2*-mutant cancers given the lack of apparent HRD. As sequencing of tumors has become more common and our understanding of synthetic lethal approaches in the context of HRD continues to evolve, it will become increasingly critical to understand the functional consequences of genomic and sporadic mutations on functional HRD. Thus, it stands to reason that this approach may serve as a biomarker to identify PVs that exhibit functional HRD and thus potentially benefit from PARPi or other therapies that exhibit synthetic lethality with HRD.

## Data Availability

No new data were generated or analyzed for this editorial.
